# Memory-specific correlated neuronal activity in higher-order auditory regions of a parrot

**DOI:** 10.1038/s41598-020-80726-y

**Published:** 2021-01-15

**Authors:** Ryohei Satoh, Hiroko Eda-Fujiwara, Aiko Watanabe, Yasuharu Okamoto, Takenori Miyamoto, Matthijs A. Zandbergen, Johan J. Bolhuis

**Affiliations:** 1 Department of Physiology, Kitasato University School of Medicine, Kanagawa, Japan; 2 Graduate School of Human Arts and Sciences, University of Human Arts and Sciences, Saitama, Japan; 3 Laboratory of Behavioral Neuroscience, Department of Chemical & Biological Sciences, Japan Women’s University, Tokyo, Japan; 4 Department of Psychology, Japan Women’s University, Kanagawa, Japan; 5 Department of Psychology, Cognitive Neurobiology and Helmholtz Institute, Utrecht University, Utrecht, The Netherlands; 6 Department of Psychology, University of Cambridge, Cambridge, UK; 7 St. Catharine’s College, University of Cambridge, Cambridge, UK

**Keywords:** Auditory system, Learning and memory, Sensorimotor processing

## Abstract

Male budgerigars (*Melopsittacus undulatus*) are open-ended learners that can learn to produce new vocalisations as adults. We investigated neuronal activation in male budgerigars using the expression of the protein products of the immediate early genes *zenk* and *c-fos* in response to exposure to conspecific contact calls (CCs: that of the mate or an unfamiliar female) in three subregions (CMM, dNCM and vNCM) of the caudomedial pallium, a higher order auditory region. Significant positive correlations of Zenk expression were found between these subregions after exposure to mate CCs. In contrast, exposure to CCs of unfamiliar females produced no such correlations. These results suggest the presence of a CC-specific association among the subregions involved in auditory memory. The caudomedial pallium of the male budgerigar may have functional subdivisions that cooperate in the neuronal representation of auditory memory.

## Introduction

The ability to learn vocalisations by imitation is a rare trait in the animal kingdom. It is absent in non-human primates, but present in humans, certain marine mammals, and three avian taxa (songbirds, parrots, and hummingbirds^1–3^). Thus, vocal learning in songbirds and parrots has become a prominent animal model for human speech and language^1, 4–10^. Vocal production learning enables complex communication in both humans and birds^11^. Furthermore, the brain regions involved in auditory-vocal learning in birds are analogous to those important for producing and processing speech in humans^1^.

The expression of immediate early genes (IEGs) is often used as a marker of neuronal activation^12^ and has been helpful in mapping neural activation during the production and perception of vocalisations in birds^6, 8,10,13^. An IEG, *zenk* (an acronym of *zif-268*, *egr-1*, *ngfI-a*, and *krox-24*) and its protein product (Zenk) have been used to investigate the effects of exposure to conspecific vocalisations on neuronal activation in the caudomedial mesopallium (CMM) and caudomedial nidopallium (NCM; secondary auditory regions) in songbirds^14–17^ and parrots, especially the budgerigar (*Melopsittacus undulatus*)^4,6,18–20^. Within the avian pallium, Field L2 receives auditory connections from the thalamus and in turn projects onto Fields L1 and L3, which project to higher-order auditory regions (the CMM and NCM)^1^. In songbirds, these secondary auditory regions are a substrate of auditory memory that is formed during song learning^8–10,21^. As such, these higher-order auditory regions have been suggested to be the avian equivalent of the human auditory association cortex in the temporal lobe, including Wernicke's area^1,8^. While Zenk and Fos (another IEG protein product, *c-fos*) are potent neural markers for identifying brain regions associated with learning and memory^22^, there are differences in the signaling cascade that regulate them^22^. Zenk is closely involved in the activation of N-methyl-D-aspartate (NMDA) receptors^23^ and is essential for the induction of long-term potentiation (LTP) in the hippocampus and formation of long-term memories in rodent spatial and non-spatial learning^23,24^. A study in rats revealed that inhibition of the NMDA receptors with the antagonist MK-801 disrupted spatial memory encoding and attenuated expression of Zenk (Zif268) in the hippocampus, but not Fos, indicating a strong association between NMDA receptor activation and Zenk (but not Fos) expression in the hippocampus during spatial learning^25^. As shown in the study^25^, differential expression of Zenk and Fos implies gene- and region-specific functions of these IEGs in mammalian brains^26–31^. A study in zebra finches (*Taeniopygia guttata*), the most widely studied songbird species, reported differential expression of Zenk and Fos following conspecific song stimulation in higher-order auditory regions^32^. In male zebra finches reared with conspecific birds in communal aviaries, the density of Zenk-positive neurons, but not Fos-positive neurons, was increased in the CMM/NCM of males following exposure to zebra finch songs at post-hatching day 30 (phd 30)^32^. In contrast, in female zebra finches, who do not sing, the density of Fos-positive neurons, but not Zenk-positive neurons, was increased in the CMM/NCM^32^. In line with the IEG study, the study using pharmacological inhibitor^21^ showed the formation of an auditory memory of tutor song requires the extracellular signal-regulated kinase (ERK) signaling pathway in a brain area including the CMM/NCM of male zebra finches. The induction of Zenk is regulated by the ERK in the zebra finch auditory forebrain^33^.

**Figure 1:**
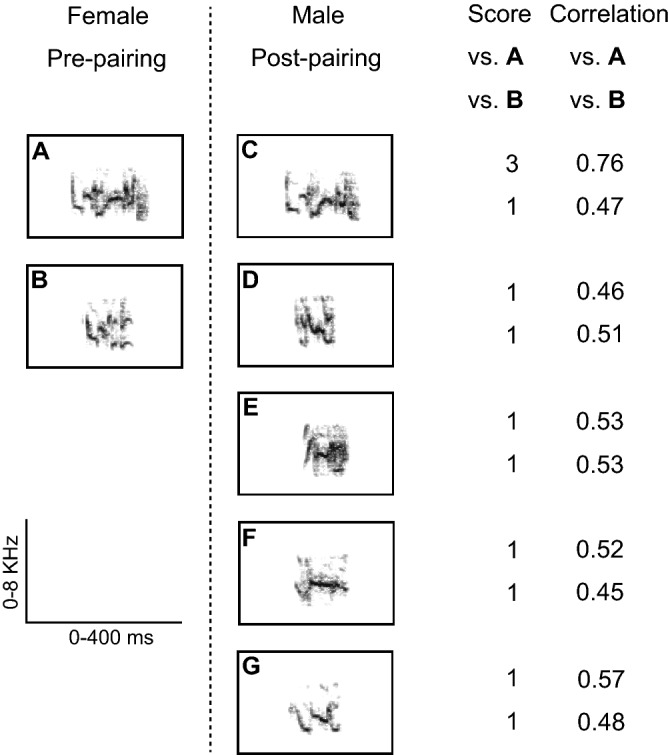
Sonograms of CCs. The female pre-pairing (left column) had two types of CC (**A**, **B**), and the male at 5 weeks post-pairing (right column) had five types of CC (**C**–**G**). We randomly selected five exemplar sonograms of each CC type and used them for rating the CC Similarity Score (Score). In each of 10 pairwise comparisons, the CC Similarity Score was rated as 1 (no similarity), 2 (modest similarity), or 3 (good similarity). Only type C was rated as good similarity and had a cross-correlation value (Correlation) greater than 0.75. In cross-correlations between two CCs from different CC types, values greater than 0.75 are highly unlikely to occur by chance^38^. Type C was not present in the CC repertoire of male pre-pairing.

Parrots learning their contact calls (CCs) may be a good animal model for human speech acquisition^34, 35^. Both sexes of the budgerigar produce individually distinct CCs (Fig. 1). At any given point in time, a bird may have a repertoire of one to several different types of CCs. CC production and recognition learning of both sexes of the budgerigar are observed in the laboratory^36,37^. During pair bond formation, males learn to produce the CCs of females and thus create a new CC type^38–40^, and also learn to recognise the CCs of their mate^20^. CC production learning reliably occurs in males paired with females, compared to males in a small group of the same sex^38^. In parrots, few studies have investigated IEG expression in response to CCs in the CMM or NCM. However, IEG responses to CC stimuli have been observed in a region of the caudomedial pallium of the budgerigar, which has been termed 'Field L' by Brauth et al.^6,18,41,42^ and 'NCM' in other studies^4,19,20^. Only one study in budgerigars has reported Zenk responses to CC stimulation in the CMM of parrots^20^. Although the caudomedial pallium in songbirds is thought to at least partly contain the neuronal substrate of memory of the tutor song, little is known about neuronal activation related to memory for mate CC in the caudomedial pallium of parrots. We paired male budgerigars with a female for 5 weeks, during which males copied the CCs of their mates. Here, we examined whether exposing male budgerigars to previously memorized CCs results in increased IEG expression, especially differential expression of Zenk and Fos, in the caudomedial pallium. Moreover, correlations of Zenk expression between regions of the caudomedial pallium have also been observed in previous^43^ and latest^44^ female songbird reports. In the Zenk study on the neural processing of anuran acoustic communication signals, the differential neural representation of conspecific and heterospecific signals involves both changes in mean activity levels across multiple subnuclei, and in the functional correlations among acoustically active areas, at the level of the midbrain and forebrain^45^. Since the functional network organisation of the brains of vertebrates is thought to be closely related to neural processing of signal coding, we further analysed for correlations among the post-exposure IEG expressions of the CMM and two NCM subregions of the caudomedial pallium.

## Results

### Contact call (CC) learning

In a representative pair, a male had five types of CCs after pairing, and one type of them showed a high 'CC Similarity Score' by visual inspection (score 3) and a 'Maximum Cross-correlation Value' (0.76; see “Methods”) with the mate CC prior to pairing (Fig. 1). The CC Similarity Score for all the experimental males after pairing (2.4 ± 0.02) was significantly greater than that for the same males prior to pairing (1.0 ± 0.00; *P* < 0.05, Wilcoxon's matched pairs test). In addition, the mean Maximum Cross-correlation Value between the male CCs after pairing and the mate female CCs before pairing was 0.73 ± 0.02. The Value in these 'within-pair' comparisons is significantly higher than that ('extra-pair' comparisons) between the male CCs after pairing and the other 11 female CCs, which was 0.65 ± 0.01 (*P* < 0.01, Wilcoxon's matched pairs test). That is, males had copied the CCs of their mates within 5 weeks after being paired with them, and the CC learning of males was confirmed. The mean CC Similarity Scores were not significantly different among the three experimental groups (Kruskal–Wallis test, H = 1.56; mean Similarity Scores ± SEM: 2.6 ± 0.26 in the MATE group; 2.4 ± 0.26 in the UNFAMILIAR group; 2.1 ± 0.34 in the SILENCE group).

### IEG expression

**Figure 2:**
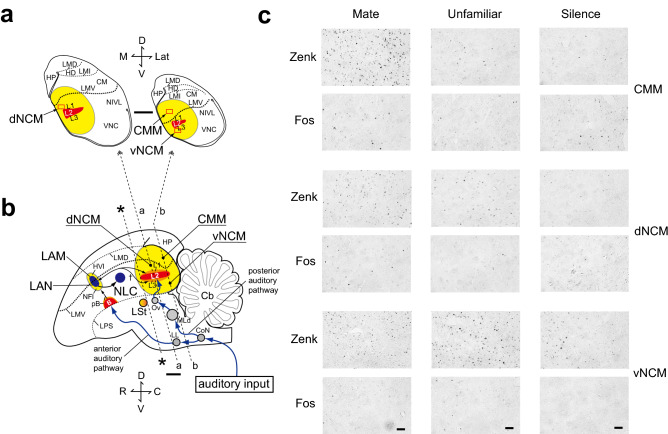
Zenk and Fos expressions in response to CCs in the budgerigar brain. (**a**) Coronal sections cut at level 'a' and 'b' in (**b**). Rectangles in the sections represent the counting frames. (**b**) A schematic drawing of a parasagittal plane in the auditory system. The caudal-most part of the LSt was defined as coordinates zero (*). Yellow regions indicate the caudomedial pallium (**a**, **b**) and a projection field (HVl/NFl) of the auditory B surrounding the LAM/LAN (**b**). Caudal and rostral lines shown in blue arrows in (**b**) indicate anterior and posterior auditory pathways, respectively. †; Reciprocal structural connections from the results of Farabaugh and Wild^64^. Lat, Lateral; Ros, Rostral; D, Dorsal; V, Ventral; C, Caudal; M, Medial. (**c**) Photomicrographs of coronal sections of the budgerigar brain showing Zenk- and Fos-immunoreactivity. See text for details. Scale bar represents 1 mm in (**a**, **b**) and 50 μm in (**c**). Anatomical Abbreviations: B, Basorostralis; Cb, Cerebellum; CM, Caudal mesopallium; CMM, Caudomedial mesopallium; CoN, Cochlear nucleus; HD, Densocellular part of the hyperpallium; HP, Hippocampus; HVC, A letter-based name, a vocal nucleus in songbirds (no abbreviation); HVl, Lateral ventral hyperstriatum (former name); L1, L2, L3, Subdivisions of Field L complex; LAM, Lateral nucleus of the anterior mesopallium; LAN, Lateral nucleus of the anterior nidopallium; LL, Nucleus of the lateral lemniscus; LMD, Lamina mesopallium dorsale; LMI, Lamina mesopallium intermediate; LMV, Lamina mesopallium ventrale; LPS, Lamina pallio-subpallialis; LSt, Lateral striatum; MLd, Dorsal part of the lateral mesencephalic nucleus; NC, Caudal nidopallium; NCM, Caudomedial nidopallium; dNCM, Dorsal caudomedial nidopallium; vNCM, Ventral caudomedial nidopallium; NIVL, Ventral lateral intermediate nidopallium; NFl, Lateral frontal neostriatum (former name); NLC, Central nucleus of the lateral nidopallium; Ov, Nucleus ovoidalis of the thalamus; pB, Peri-basorostralis; VNC, Caudoventral nidopallium.

Figure 2c shows representative photomicrographs of Zenk- and Fos-immunoreactive (-IR) cell nuclei in the CMM, dorsal NCM (dNCM), and ventral NCM (vNCM) (see “Methods”). In the CMM and dNCM, Fos-IR cell nuclei were observed in the SILENCE group but not in the vNCM. Two-way repeated-measures ANOVA (Stimulus [MATE, UNFAMILIAR, or SILENCE] as between-subjects factor, Zenk and Fos as within-subjects factor) revealed significant effects of both Stimulus (F_2,33_ = 12.660, *P* < 0.001) and IEG (F_1,33_ = 124.628, *P* < 0.001). There was no significant interaction between Stimulus and IEG (F_2,33_ = 0.741, *P* = 0.484). We therefore analysed Zenk and Fos expression data separately.

### Zenk expression

**Figure 3:**
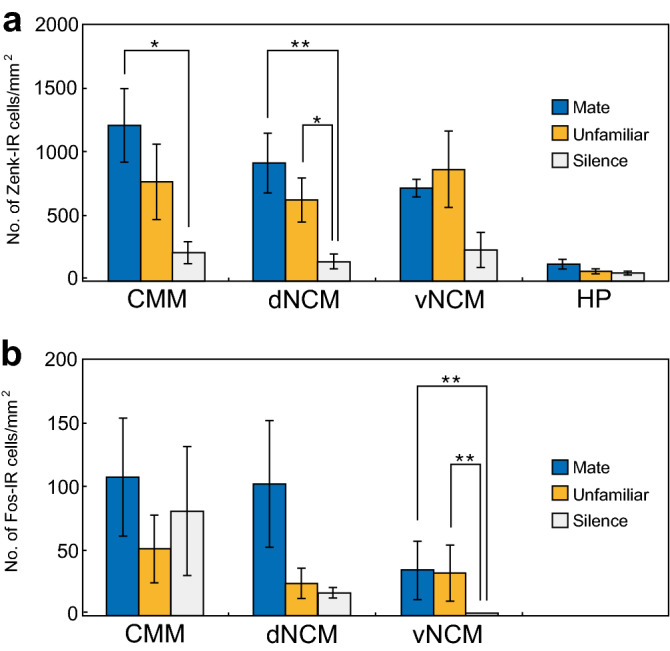
Differential patterns of Zenk and Fos expression in response to CCs in the caudomedial pallium. Mean (± SEM, *n* = 4) number of IEG-IR cells per square millimetre in the CMM, dNCM, vNCM, and hippocampus (HP) in Zenk expression (**a**) and in the CMM, dNCM, and vNCM in Fos expression (**b**). Statistical significance is indicated by the asterisks (*P < 0.05; **P < 0.01).

Zenk expression data in the hippocampus (HP) were available as a control region (see “Methods”). Two-way ANOVA (Stimulus [MATE, UNFAMILIAR, or SILENCE], and Brain Region [CMM, dNCM, vNCM, and HP] are factors) revealed significant effects of both Stimulus (F_2,36_ = 16. 350, *P* < 0.001) and Brain Region (F_3,36_ = 14.631, *P* < 0.001). There was no significant interaction between Stimulus and Brain Region (F_6,36_ = 0.662, *P* = 0.680). The data were then analysed separately for each of subregion (CMM, dNCM, vNCM, or HP) with one-way ANOVA with Stimulus again the between-subjects factor. We found a significant effect of Stimulus in Zenk expression levels in the CMM (F_2,9_ = 7.788, *P* = 0.014) and dNCM (F_2,9_ = 9.197, *P* = 0.007). Post-hoc Bonferroni testing revealed a significant difference between the MATE and SILENCE groups in the CMM (*P* < 0.05, Fig. 3a). In the dNCM, significant differences were observed between the MATE and SILENCE groups (*P* < 0.01), and between the UNFAMILIAR and SILENCE groups (*P* < 0.05, Fig. 3a). One-way ANOVA revealed a significant effect of Stimulus in the vNCM (F_2,9_ = 4.263, *P* = 0.049), but Bonferroni testing did not reveal any significant difference between groups. One-way ANOVA revealed no significant effect of Stimulus in the HP (F_2,9_ = 3.956, *P* = 0.058). There were no significant differences between MATE and UNFAMILIAR groups in all brain regions. There was no significant correlation between the CC Similarity Score and number of Zenk-IR cells for birds of the MATE group (*n* = 4), UNFAMILAR group (*n* = 4), or SILENCE group (*n* = 4) in the CMM, dNCM, vNCM, or HP (Spearman's rank correlation test).

### Fos expression

As two-way ANOVA (Stimulus and Brain Region are factors) indicated significant effects of both Brain Region (F_2,27_ = 5.721, *P* < 0.01) and Stimulus (F_2,27_ = 5.312, *P* < 0.05), and there was no significant interaction of Stimulus and Brain Region (F_4,27_ = 1.644, *P* = 0.192), the data for each of the three brain subregions (CMM, dNCM, and vNCM) were further analysed. One-way ANOVA indicated no significant effect of Stimulus on Fos expression levels in the CMM (F_2,9_ = 0.501, *P* = 0.622) or dNCM (F_2,9_ = 1.898, *P* = 0.205). A significant effect of Stimulus on Fos expression levels in the vNCM (F_2,9_ = 10.058, *P* = 0.005) was detected. Bonferroni testing revealed significant differences in Fos expression levels in the vNCM between the MATE and SILENCE groups (*P* < 0.05), and between the UNFAMILIAR and SILENCE groups (*P* < 0.05, Fig. 3b). There were no significant differences between MATE and UNFAMILIAR groups in all brain regions. We observed that the basal expression (i.e., the expression in the SILENCE group) of Fos was very low in the vNCM (Fig. 3b). There was no significant correlation between the CC Similarity Score and number of Fos-IR cells for birds of the MATE group, UNFAMILAR group, or SILENCE group in the CMM, dNCM, or vNCM (Spearman’s rank correlation test).

### Correlations among brain regions in Zenk activation

**Figure 4:**
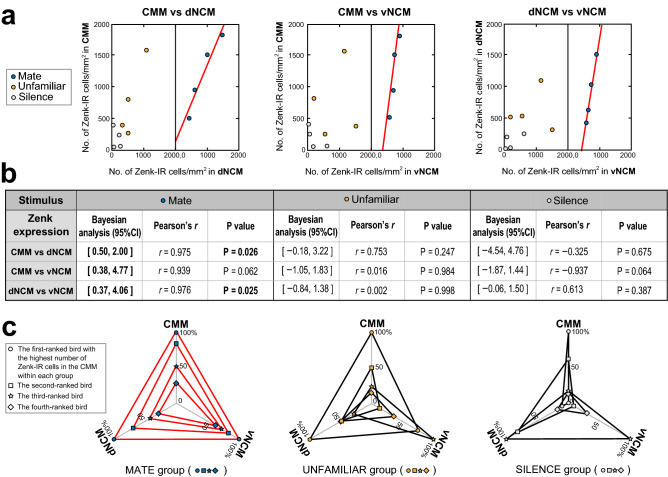
Analysis of correlations in Zenk expression between subregions of the caudomedial pallium after CC stimulation. (**a**) Scatterplots of number of Zenk-IR cells per square millimetre activated in response to mate CCs (right side of each figure), or unfamiliar CCs, or silence (left side of each figure) are plotted for individual subjects. Shown are correlations in CMM versus dNCM (left), CMM versus vNCM (centre), and dNCM versus vNCM (right). There were three significant linear relationships between subregions with Bayesian analysis, where linear regression lines in red are fitted. (**b**) The statistics of the data in (**a**) are given in the table with CIs of Bayesian analysis, correlation coefficients (*r*) and *P* values of Pearson's correlation analysis. Statistically significant values are highlighted. (**c**) Shown are patterns of correlation across subregions of the number of Zenk-IR cells activated in response to mate CC (left), unfamiliar CC (centre), or silence (right). Within a group, the expressions were scaled to the data of the bird with the highest Zenk expression in each of the three subregions, and the CMM, dNCM, and vNCM values for the same subject are plotted as percentages and connected with lines. The values of the first-ranked bird in the CMM within each group are indicated by circles in the figure. The values of the second-, third-, and fourth-ranked bird in the CMM within each group are indicated by squares, stars, and diamonds, respectively. The types of stimuli are color-coded as in (**a**). Note that only in the case of mate CC (left) was a well-organised and ordered 'triangle' relationship observed within the group. In the other cases, i.e., unfamiliar CC (centre) or silence (right), these triangles overlap irregularly or do not form. Inter-subregion lines drawn in red indicate significant linear relationships between subregions with Bayesian analysis.

Figure 4a shows scatter plots of the numbers of Zenk-IR cells (Zenk expressions) in each pair of subregions and b summarises all statistical values for the relationships among the subregions. In the MATE group, we observed significant positive correlations between Zenk expressions in the CMM vs dNCM (*r* = 0.975, *P* = 0.026), and in the dNCM vs vNCM (*r* = 0.976, *P* = 0.025), and a marginally significant positive correlation in the CMM vs vNCM (*r* = 0.939, *P* = 0.062). In the UNFAMILIAR group, we observed no significant correlations in Zenk expression (CMM vs dNCM, *r* = 0.753, *P* = 0.247; CMM vs vNCM, *r* = 0.016, *P* = 0.984; and dNCM vs vNCM, *r* = 0.002, *P* = 0.998). Likewise, we observed no significant correlations in Zenk expression in the SILENCE group (CMM vs dNCM, *r* = ** − **0.325, *P* = 0.675; CMM vs vNCM, *r* = ** − **0.937, *P* = 0.064; and dNCM vs vNCM, *r* = 0.613, *P* = 0.387). Figure 4c shows the differential pattern of Zenk expression across subregions in each of the three Stimulus groups. The pattern of Zenk expression in each subregion per subject showed an ordered relationship in the MATE group, like a nested 'triangle', whereas the UNFAMILIAR and SILLENCE groups did not show such relationships. In the MATE group, the rank order of Zenk expression across subjects did not vary across subregions, suggesting that Zenk in the three subregions was expressed at approximately the same rate per subject.

In order to examine the relationship between the Zenk expressions in each pair of subregions (i.e., CMM, dNCM, and vNCM), we also performed Bayesian analyses with a simple regression model, using the data for the correlation analyses mentioned above (Fig. 4). For the all three pairs of subregions in the MATE group, the 95% confidence interval (CI) for the regression coefficients did not include zero and the coefficients were positive values. These results indicate that for an increase in the Zenk expression of one of the subregions, the other subregion also increased in the expression for each pair of subregions in the MATE group. However, for the all three pairs in the UNFAMILIAR group and those in the SILENCE group, the CIs for the regression coefficients included zero. These results indicate that there was no linear relationship between the Zenk expressions in the UNFAMILIAR or the SILENCE group.

### Correlations among brain regions in Fos activation

**Figure 5:**
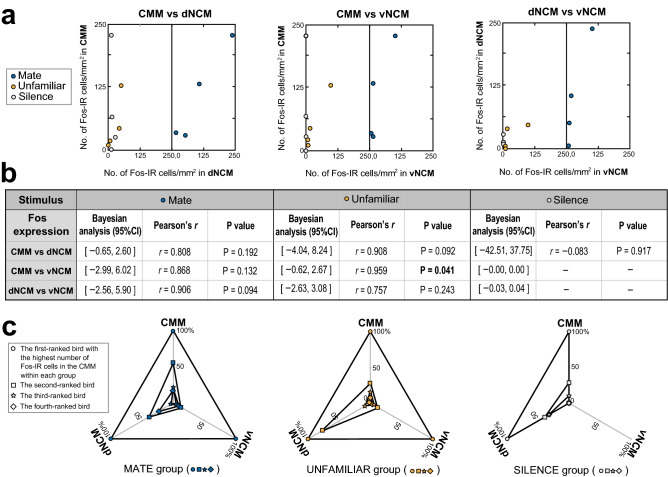
Analysis of correlations in Fos expression between subregions of the caudomedial pallium after CC stimulation. Shown are data with the same conventions as in Fig. 4 but pertaining to the Fos-immunopositive cell counts. Unlike the results revealed by Zenk expression (Fig. 4), there was no linear relationship between subregions for the MATE, the UNFAMILIAR or the SILENCE group with Bayesian analysis.

Figure 5 gives the Fos data corresponding to Fig. 4. Figure 5a shows scatter plots of the numbers of Fos-IR cells for each pair of regions and b reports the statistical values. In the MATE group, we observed no significant correlations (CMM vs dNCM, *r* = 0.808, *P* = 0.192; CMM vs vNCM, *r* = 0.868, *P* = 0.132; and dNCM vs vNCM, *r* = 0.906, *P* = 0.094). In the UNFAMILIAR group, we observed a significant positive correlation between Fos expressions in the CMM and those in the vNCM (*r* = 0.959, *P* = 0.041), but not in the other two pairs of subregions (CMM vs dNCM, *r* = 0.908, *P* = 0.092; and dNCM vs vNCM, *r* = 0.757, *P* = 0.243). In the SILENCE group, we observed no significant correlation between Fos expressions in the CMM and dNCM (*r* = ** − **0.083, *P* = 0.917). Pearson's *r* was undefined for the other two comparisons in this group because of zero values of expression in the vNCM. Figure 5c shows the differential pattern of Fos expression across subregions in each of the three Stimulus groups. In contrast to Zenk expression, pairwise correlations between Fos expressions were significant only in CMM versus vNCM in the UNFAMILIAR group. No ordered relationship, which forms a nested triangle, was observed in any of the three groups.

In order to examine the relationship between the Fos expressions in each pair of subregions (i.e., CMM, dNCM, and vNCM), we also performed Bayesian analyses with a simple regression model, using the data for the correlation analyses mentioned above (Fig. 5). For the all three pairs in each of the three conditions (the MATE, the UNFAMILIAR and the SILENCE groups), the CIs for the regression coefficients included zero. These results indicate that there was no linear relationship between the Fos expressions for the MATE, the UNFAMILIAR or the SILENCE group.

## Discussion

In the present study, male budgerigars mimicked the acoustic structure of the CCs of their female mates within 5 weeks after being paired (Fig. 1). These behavioural results are consistent with previous findings by Hile et al*.*^38,40^ and thus confirm that male budgerigars learn the CCs of their mates. We investigated neuronal activation based on Zenk and Fos expression in response to female CCs in the caudomedial pallium of male budgerigars. In the CMM/dNCM, CC stimulation led to significantly increased Zenk expression when compared to silence. A trend to increase was observed in the vNCM, while no increase was detected in the hippocampus (Fig. 3a). In contrast, increased Fos expression in response to CC was observed in the vNCM but not in the CMM or dNCM (Fig. 3b). The differential pattern of IEG expression suggests a functional difference between the CMM/dNCM pair and the vNCM. For both IEGs, we found no significant differences in mean expression levels between the MATE group (mate CC stimuli) and the UNFAMILIAR group (unfamiliar CC stimuli) in any brain region (Fig. 3a, b). However, only in the MATE group did Zenk (but not Fos) expression reveal significant positive correlations in neuronal activation among subregions (Fig. 4). In the UNFAMILIAR group, in contrast, we observed no significant correlations of neuronal activation among the subregions. In addition, only the mate CC stimuli induced an across-subjects pattern of Zenk expression that was consistent across subregions (Fig. 4c). These results suggest that the correlated neuronal activity among these subregions is associated with CC memory, because the context difference between the two stimuli consisted of being related or not related to this memory.

Interestingly, our findings can be interpreted as the memory of the CC not being represented by the amount of subregion activity alone, as estimated by IEG expression, but by the correlations in activity among these subregions. A design focusing only on IEG expression within the subregions would not have detected these interrelationships. Whether a correlation is observed could depend on the presence of neuronal activation that is synchronous across subregions^46^. By observing significant correlations of Zenk expression between subregions (Fig. 4), it can be inferred that the functional connectivities^45^ between these subregions lead to synchronous activity enhancements in response to CC stimuli^47^. CC memory formation may require changes in the functional connectivities or circuits among the subregions which are constituted by Zenk-positive neurons^12,48^. This is consistent with Hebb's law and cell assemblies^49^, which posits that memory formation is due to plastic changes in synaptic connections in memory-related neural circuits^50^. We do not know any direct projections among the subregions in the caudomedial pallium of the budgerigar. Further research is necessary to investigate anatomical pathways within the caudomedial pallium, although audio-vocal pathways have been found in this species (discussed below).

A study of gene expression profiles in avian brains indicated that the mesopallium cell populations containing the CMM and nidopallium cell populations containing the NCM constitute functional columns with the same physiological functions, similar to the layered structure of the mammalian cerebral cortex^51^. The CMM and NCM are structurally separated by the lamina mesopallium ventrale (LMV) in the budgerigar, as in other avian species (see Fig. 2a, b). The dNCM of budgerigars examined in this study is the region located between the LMV and Field L2. There are differences in the orientation of Field L2 across avian species, with Field L2 having a more vertical orientation and the CMM shifted more rostrally in songbirds compared to parrots^52^. In songbirds, there is limited NCM space between the LMV and Field L2. The vNCM of the budgerigar are anatomically located on the ventral side of Field L complex, while the CMM/dNCM are located on the dorsal side of Field L complex. The vNCM, but not the CMM/dNCM, exhibited Fos expression in response to CC stimulation. Further, the budgerigar vNCM is characterised by lower basal expression of Fos than that of Zenk^42^ (Fig. 3b). Based on these observations, the vNCM may be structurally or functionally different from the CMM/dNCM. The CMM/dNCM and the vNCM may cooperatively constitute a posterior auditory column^51^ of the budgerigar.

A previous study suggested a role of the CMM in CC perception in the budgerigar^20^, and the involvement of the CMM/NCM in song perception in budgerigars has also been reported^19^. A large body of evidence, much of it based upon gene expression studies, suggests that the caudomedial pallium of songbirds is involved in memory as well as perception of song^10^. In songbird studies, a representation of the auditory memory of the tutor song has been distinguished from that of the motor memory of the bird's own song (BOS)^8^. In adult zebra finches, IEG expression in the NCM was positively correlated with the strength of song learning in the males that were exposed to the tutor song, not in those to the BOS or to novel conspecific song^53^. By contrast to the NCM, IEG expression in the HVC (used as a proper name), which is a songbird nucleus involved in song production and sensorimotor learning, was positively correlated with the strength of song learning in the males exposed to the BOS, not in those to the tutor song or to novel song^10^. In the present study, the correlated neuronal activity among subregions of the caudomedial pallium was suggested to be memory-related in male budgerigars. Whether the correlated IEG expression is related to the auditory memory or to the motor memory could be tested by exposing male subjects to the bird's own call. Otherwise, another test could be considered. Male budgerigars learn to recognise their mate CC during the pairing period of 1 month^20^. In the present study, we did not conduct the preference test^37^ for mate CC after the pairing period. If the correlated IEG expression is related to the auditory memory, the IEG expression is correlated with the strength of CC learning, measured as a preference score for the mate CC.

The design of this study has a limitation in that only one type of CC stimulus can be used for a given subject. However, using a cellular compartment analysis of temporal activity based on data from fluorescence in situ hybridization (catFISH), such as *Arc* catFISH, it may be possible to provide a subject with two CC stimuli representing different contexts^54^. In the future, it will be necessary to use optogenetic methods^55^ to deepen our understanding of the mechanism of CC memory formation, since these methods can identify memory circuits by detecting IEG expression.

Activation of NMDA receptors contributes to the activation of ERKs and Zif268 (Zenk), which are essential components of a signaling cascade required for the LTP and long-term memory formation^23^. A recent study in rodents indicated a strong association between NMDA receptor activation and Zif268 (but not Fos) expression in the hippocampus during spatial learning^25^. Several studies in songbirds have examined the role of signaling cascade in song production, sensorimotor learning, and adult song plasticity. The NMDA receptors and ERK in the caudomedial pallium of the zebra finch contributes to the induction of Zenk and are essential for song learning in young birds^21,33^. The NMDA receptor subunits NR2A and NR2B show higher and lower expression, respectively, in most nuclei of the song system relative to surrounding brain subdivisions in which nuclei of the song system are located^56^. Basham et al.^57^ inactivated the lateral magnocellular nucleus of the anterior nidopallium, a song system nucleus, via injections of an NMDA receptor antagonist on days that birds were exposed to tutor song. Song learning was significantly impaired in birds that received the experimental treatment, although it is not clear whether NMDA receptor antagonist infusions affected sensorimotor integration or the formation of auditory memory^10^. The three avian groups of vocal learners have overlapping but distinct cerebral nuclei for vocal production and learning, commonly called the 'song system'. In the budgerigar, the NMDA receptor subunits NR2A and NR2B show an expression pattern similar to that of songbirds in most nuclei of the song system^58^. These receptor subunits do not seem to be differentially expressed in the caudomedial pallium compared to surrounding brain subdivisions^56^. Further confirmation of the involvement of NMDA receptors^58^, ERK and Zenk in CC learning in parrots will be needed. In visual imprinting in chicks, NMDA receptors in higher visual regions of the telencephalon are also implicated in learning. The neural circuits responsible for visual imprinting in chicks reside in the pallial layers of the telencephalon^59,60^. A subsequent study reported that NR2B-containing NMDA receptors (NR2B/NR1) in this region were essential for imprinting^61^.

In this study, we have shown that the caudomedial pallium (i.e., the posterior auditory column^51^) of the budgerigar may contain the neuronal substrate of CC memory, the basis of CC learning. However, the details of how auditory information processed through the posterior auditory column is used for CC learning remain unknown. In male songbirds that are learning vocalisations, there is continual interaction between the song system and caudomedial pallium, similar to the interaction between Broca's and Wernicke's areas in human infants acquiring speech and language. Although the budgerigar learns both songs and CC, their song system has been termed a vocal control system because research exploring the neural basis of vocal learning has mainly been performed with CC learning. Unlike songbirds, the vocal control system of budgerigars employs two structural connections, one from the caudomedial pallium and another from the anterior auditory pathway (see Fig. 2b)^4,18, 62–65^. The basorostralis (B, auditory B) located in the rostral telencephalon receives input from the intermediate nucleus of the lateral lemniscus and constitutes the anterior auditory pathway that includes the NFl (the lateral frontal neostriatum, former name) as a projection field of the auditory B^4, 7,18,62,66^ (Fig. 2b). Information from the NFl is conveyed to the adjacent HVl (the lateral ventral hyperstriatum, former name) and to the central nucleus of the lateral nidopallium^67^ (NLC), of the vocal control system^7,18^. The NLC receives auditory input from the caudal pallium via the NFl, which also receives axonal input from the auditory area in the caudal nidopallium. Plummer and Striedter^7^ suggested that auditory information from the anterior auditory pathway plays an important role in CC learning because the destruction of HVl/NFl interferes with CC learning of budgerigars but does not disturb the production of learned CCs. The HVl/NFl is a large area which has been characterised using electrophysiological and tracing methods; nevertheless, its boundaries are less clearly delineated than those of the vocal control nucleus, NLC^7, 18,62^. IEG studies have indicated that nuclei showing singing-driven Zenk expression are embedded within the HVl/NFl, and these nuclei are termed the lateral nucleus of the anterior mesopallium and lateral nucleus of the anterior nidopallium (LAN, see Fig. 2b)^4,68^. The LAN corresponds to the songbird's nuclear interfacialis of the nidopallium (NIf)^69,70^. The songbird's NIf is an important nucleus that mediates auditory and vocal representations^70,71^ and is embedded in the caudomedial pallium, whereas the LAN of the budgerigar is embedded in the anterior auditory system^68,72^. Recently, Chakraborty et al.^72,73^ reported that the vocal control system of parrots, unlike the song system of songbirds, has a duplicated anterior vocal control system (shell song system) which was thought to have evolved from the adjacent motor learning circuit^68^ in parrots^72,73^. A recent review suggests that the shell song system which does not exist in songbirds characterises an adult CC learning in parrots as open-ended learners^74^. Taken together with our results, the anatomy of the two auditory input systems from the caudomedial pallium and anterior auditory pathway to the vocal system, as well as that of the shell song system^72,73^, suggests a mechanism for organising a dynamic functional connectivity during CC learning; these structures may function in an integrated manner as the source of the highly developed CC learning ability found in budgerigars.

In summary, we have found evidence suggesting that the neuronal representation of auditory (CC) memory in male budgerigars is a functional connectivity between the subregions of the caudomedial pallium. The most important result is that exposure to the mate, but not unfamiliar CCs, induced correlated Zenk responses among subreigons in the caudomedial pallium of male budgerigars. The correlated activities among these subregions as revealed by Zenk expression and induced by stimuli differing in relatedness to CC memory suggest organisation of functional connectivity among the subregions. The caudomedial pallium of the male budgerigar may therefore be functionally subdivided and play an important role in the neuronal representation of CC memory as cooperativity among subdivisions.

## Methods

### Subjects

All experimental procedures were performed in accordance with Japanese law and approved by the Animal Experiments Committee of Japan Women's University (Permit Number: II07-13). The 24 budgerigars (12 males and 12 females) in the present experiment had been used in a previous study^37^ that only assessed female behaviours. All birds were obtained from a local supplier and kept in a controlled environment suitable for breeding (23 ± 3 °C and a light/dark cycle of 14:10 h light:dark) throughout the study, as described previously^37^. Hile et al*.*^38^ developed a behavioural paradigm to elicit vocal imitation reliably and fast (within an average of 2.1 weeks) in adult male budgerigars. We used this paradigm in the present experiment. Males and females were acquired from different breeders and were housed in separate rooms, so that females were unfamiliar with males prior to the study. Birds were initially singly housed, and their vocalisations were recorded. After recording of all birds was completed, pairing was initiated. As described by Hile et al*.*^38^, pairs were prevented from seeing other pairs in the same room by means of wooden barriers; however, they were in vocal contact with other pairs. Hile et al*.*^38^ reported the shared calls emerged within 3 days to 5 weeks after pairing. After the pairing period of 5 weeks, each bird was isolated from the mate and kept in an individual cage, and their vocalisations were recorded again. To assess the development of pair bonds, we performed behavioural observations on days 2 and 12 after the pairs were placed into breeding cages. As reported in the previous study^37^, (1) the birds showed significantly more allopreening (a behaviour associated with pair bonding in the budgerigar) on day 12 than on day 2, and (2) six of 12 females laid eggs by day 24 after the start of pairing.

### Recording and analysis of CCs

At any given point in time, a bird has a repertoire of one to several different patterns or types of CC^36, 38^. We recorded and analysed the CC repertoires of all birds. The methods used to record and analyse CCs were similar to those described in previous studies^20,37,38^. We recorded at least 80 CCs per bird at each time point. After classifying CCs into types, we paired birds dissociatively with respect to CCs, that is, pair members did not have similar CCs at this time (see below). The CCs at 5 weeks after pairing were compared with those of his assigned female (see below).

*Call classification.* According to our previous study^37^, the vocalisations were displayed as sonograms (fast Fourier transformation, FFT, size: 256, temporal resolution 3 ms) using the sound analysis software Avisoft SASLab Pro (Avisoft Bioacoustics, Berlin, Germany). Farabaugh et al*.*^36^ classified CCs on the basis of their frequency modulation patterns on sonograms. This procedure proved reliable for classifying CCs within individuals; average interobserver agreement for four independent observers was 93% in our pilot analysis conducted using 880 CCs (80 CCs recorded from each of 11 males). CCs of each bird were classified into types by an observer (one of four observers in our pilot analysis mentioned above) naïve to the identity of the birds. Of all CC types for each bird, one type occurs most frequently and is therefore termed 'the dominant CC'. We determined this CC for each bird.

*Call similarity.* Prior to pairing, all the CC types of each male were compared with those of his mate by visual inspection of sonograms. A blinded observer rated the degree of similarity as 1 (no similarity), 2 (modest similarity), or 3 (good similarity). All comparisons were rated as 1. We determined whether a male had imitated one of his paired female's CC types. Each male had several CC types (range: 5–31), while the number of CC types of each female ranged from one to five. CC types of a male at 5 weeks after pairing were compared to those of his mate prior to pairing by means of visual inspection of sonograms (Fig. 1). We randomly selected 5 exemplars of each CC type from each female prior to pairing. In the same way, 5 exemplars were randomly selected for each CC type from each male at 5 weeks after pairing. If there were fewer than 5 exemplars of a given type, all of the exemplars of that type were used. A blinded observer rated the degree of similarity as above. The highest score of all pairwise comparisons for 5 weeks after pairing was determined and defined as 'the CC Similarity Score' for each bird pair.

*Spectrogram cross-correlation analysis.* We also conducted spectrogram cross correlation, using Avisoft Correlator version 2.2 (Avisoft Bioacoustics), and verified that 'CC Similarity Score' was a reliable measure. The correlation coefficient is a value ranging from − 1 to + 1. A value of 1 means that the two sonograms are identical. A value of 0 means that there is no similarity between the sonograms. The mean value of the correlation within the same type classified by visual inspection was 0.75 ± 0.01. This is significantly higher than that among different types, which was 0.54 ± 0.02 (*P* < 0.01, Mann–Whitney U-test). CC types of a female prior to pairing were compared to those of her mate at 5 weeks after pairing, using the exemplar sonograms selected for visual inspection mentioned above. For each male, we determined the three highest correlation values in all of the comparisons between sonograms of male CCs at 5 weeks after pairing and those of his mates' prior to pairing. We defined this averaged value as 'the Maximum Cross-correlation Value'. The two methods (visual inspection and cross correlation by software) produced similar results, with higher scores by visual inspection having larger cross correlation values (mean values ± SEM: 0.75 ± 0.036 in the group with similarity score 3; 0.60 ± 0.030 in the group with similarity score 2; 0.48 ± 0.006 in the group with similarity score 1). There was a significant difference in cross correlation values among three groups with different scores by visual inspection (analysis of variance [ANOVA], F_2, 444_ = 21.819, *P* < 0.0001). There were significant differences in all three comparisons of a post-hoc Scheffe test (similarity score 3 vs similarity score 2, 3 vs 1, and 2 vs 1).

### CC playback

At 6 weeks after pairing, males were placed in a cage in a sound-attenuating chamber equipped with a speaker (AS-5; Kenwood Corp., Tokyo, Japan) for at least 12 h prior to the start of stimulus presentation. Then, each male was exposed to a recording of his mate CC (group MATE; *n* = 4), a CC of a novel adult female (group UNFAMILIAR; *n* = 4) or kept in silence (group SILENCE; *n* = 4). We used the female's pre-pairing CC type that a male had imitated as mate CCs. For group UNFAMILIAR, we prepared four CCs from four females that were unfamiliar to the males; these recordings had been made before pairing in previous pairing experiments using different birds from those used in the present study.

All playback was performed in the morning, as described previously^20^. Lights were switched off 15 min before the onset of playback. During playback, each bird was exposed to the repetition of a single CC (0.5 CC per sec) at regular intervals for 30 min. CCs were broadcasted at the peak value of 80 dB SPL (NA-14 Sound Level Meter; Rion Corp., Tokyo, Japan; A-weighting, slow response), measured 20 cm away from the speaker. The birds remained in darkness for 1 h after the end of CC playback and were then sacrificed. We monitored vocal behaviour of 24 birds during 30 min of playback. We adopted the criterion similar to that in a previous study^75^; in brief, we excluded cases in which songs were produced or more than 5% of CCs broadcasted from the speaker were produced by the subject. None of the tested 12 males vocalised the song, nor produced CCs more than 5% of those broadcasted.

### IEGs analysis

One hour after the end of exposure to the stimulus, the birds were administered an overdose of sodium pentobarbital (Nembutal, Abbott, Japan) and subsequently perfused intra-cardially with saline and a Zamboni fixative (4% paraformaldehyde in 0.1 M phosphate-buffered saline (PBS) containing 10.5% of a saturated picric acid solution). After fixation, dissected brains were divided into two coronal parts. They were cryoprotected in PBS containing sucrose and frozen and maintained at − 20 °C until cut. Frontal frozen brain sections were processed for Zenk-IR or Fos-IR cell nuclei by immunocytochemistry, as described previously^19,76^. We used polyclonal antibodies against Egr-1 (C-19, SC-189, dilution 1:15,000) or c-Fos (K-25, SC-253, dilution 1:15,000) raised in rabbit (Santa Cruz Biotechnology, Santa Cruz, CA, USA). Adjacent sections were Nissl stained to enable identification of anatomical markers.

### Image analysis

In songbirds and parrots, Field L2 in the caudal telencephalon (analogous to the mammalian primary auditory cortex) receives input from the thalamic auditory nucleus ovoidalis (Ov) and constitutes a posterior auditory pathway (system) that includes Fields L1 and L3, the NCM, and CMM^4, 41,77^ (Fig. 2b). In budgerigars, Jarvis and Mello^4^ observed song-induced ZENK expression in the NCM and Fields L1 and L3. As the authors could not distinguish Nissl boundaries between the different nidopallial fields outside of L2, they designated the entire region that showed hearing-induced expression in the nidopallium as the budgerigar NCM, which surrounds the presumed Fields L1 and L3 (Fig. 2a, b). We were unable to distinguish the boundaries between the different nidopallial fields outside of L2 with Nissl staining, but we identified L2 and the LMV, which is a distinct boundary between the CMM and NCM^50^ (see also Fig. 2a, b). We have followed Jarvis and Mello's^4^ nomenclature and used the terms CMM and NCM^19,20,77^. In the present study, we sampled two regions within the NCM at dorsal and ventral levels (dNCM and vNCM), and CMM, comparable with those in our previous papers^19,20,76^.

We quantified the number of IEGs-IR cell nuclei (cells) in the CMM, dNCM, and vNCM. As a non-auditory-related control region (see Fig. 3a, b), we also investigated Zenk expression in the HP, as described previously^19,20,76^. The counting frames (290 × 450 μm) in the CMM, dNCM and vNCM were placed adjacent to the midline (Fig. 2a). In all cases, the counting frames were positioned by an experimenter blinded to the experimental condition of each animal. Photomicrographs of the counting frames were captured by a digital camera with 8-bit intensity resolution (256 levels of grey). We took a total of four samples from both hemispheres (two from each hemisphere) per region (CMM, dNCM, vNCM, and HP) per subject. For each region, the mean number of IEGs-IR cells were calculated for the dNCM and HP at 1.00 and 1.06 mm caudal to coordinates zero (level 'a' in Fig. 2a, b), and for the CMM and vNCM at 2.00 and 2.06 mm caudal to coordinates zero (level 'b' in Fig. 2a, b**)**. Image analysis was performed in a semiautomated manner with a PC-based system equipped with KS400 version 3.0 software (Carl Zeiss Vision, Oberkochen, Germany), as described in detail previously^76^. Counting was performed blinded to the experimental history of the subjects.

### Statistical analysis of IEGs expressions

IEG expression was analysed with analysis of variance (ANOVA) with factor Group (MATE, UNFAMILIAR, or SILENCE) and factor Brain Region. Subsequently, we used one-way ANOVAs for individual brain regions. Post-hoc comparisons were performed using Bonferroni tests. Summary of replications: 12 animals per IEG, four animals per group, and four 290 × 450 μm counting fields per brain region (two on each side) per animal. The relationship between the IEG expressions in each pair of subregion was examined by a Pearson's product-moment correlation analysis. The data were log transformed before statistical analysis to satisfy the assumptions of the parametric tests and analysed using StatView version 5 (SAS Institute Inc., Cary, NC, USA). Level of significance was set at *P* < 0.05.

In order to examine the relationship between the IEG expressions in each pair of subregions, we used a simple regression model by Bayesian analysis. Bayesian analysis was employed because maximum likelihood methods require large data sets^78^. For a regression coefficient, we calculated the 95% confidence interval (CI). If the CI of a regression coefficient (e.g. the coefficient showing the effect of dNCM on CMM) does not include zero (the value in our null hypotheses), it indicates that there is a significant linear relationship between the two variables (dNCM and CMM in this example).

## Data Availability

The datasets generated and/or analysed during the current study are available from the corresponding authors on request.
